# Supplementary material for: Molecular recognition of an odorant by the murine trace amine-associated receptor TAAR7f
Source: Nat Commun. 2024 Aug 30;15:7555. doi: 10.1038/s41467-024-51793-w (PMC11364543; doi:10.1038/s41467-024-51793-w)
Supplement: Supplementary file 1 — Supplementary Information [file 41467_2024_51793_MOESM1_ESM.pdf]

Supplementary Information

Molecular recognition of an odorant by the murine trace amine-associated receptor TAAR7f

Anastasiia Gusach, Yang Lee, Armin Nikpour Khoshgrudi, Elizaveta Mukhaleva, Ning Ma, Eline J. Koers, Qingchao Chen, Patricia C. Edwards, Fanglu Huang, Jonathan Kim, Filippo Mancia, Dmitry B. Veprintsev, Nagarajan Vaidehi, Simone N. Weyand, Christopher G. Tate

Contents

Supplementary Table 1 | Cryo-EM data collection, refinement and validation statistics

Supplementary Table 2 | Numerical values for data displayed in the bar graphs Figs 3b-d

Supplementary Figure 1 | Comparison of receptor amino acid sequences

Supplementary Figure 2 | mTAAR7f purification and cryo-EM of the mTAAR7f–mini-G_s–Nb35 complex

Supplementary Figure 3 | Flow chart of cryo-EM data processing

Supplementary Figure 4 | Amino acid sequence conservation in the OBS and activation switches of TAARs and β_2AR

Supplementary Figure 5 | MD simulation of DMCHA association to mTAAR7f

Supplementary Figure 6 | Alignment of the amino acid sequences of mTAAR7f and β_2AR

Supplementary Figure 7 | MD simulations of mTAAR7f and analysis of changes in activation switches

Supplementary Figure 8 | MD simulation of DMCHA association to mTAAR7f

Supplementary Figure 9 | Variation of backbone atom and ligand atom RMSD during MD simulations

References cited only in the Supplementary Information

57. Goujon, M. *et al.* A new bioinformatics analysis tools framework at EMBL-EBI. *Nucleic Acids Res.* **38**, W695–W699 (2010).

58. Sievers, F. *et al.* Fast, scalable generation of high-quality protein multiple sequence alignments using Clustal Omega. *Mol. Syst. Biol.* **7**, 539 (2011).

59. Altschul, S. Gapped BLAST and PSI-BLAST: a new generation of protein database search programs. *Nucleic Acids Res.* **25**, 3389–3402 (1997).

Supplementary Table 1 | Cryo-EM data collection, refinement and validation statistics

**Data collection and processing**	
Magnification	96,000x
Voltage (kV)	300
Electron exposure ($e^{-}/Å^{2}$)	55
Defocus range (μm)	-0.8 to -2.4
Pixel size (Å)	0.824
Symmetry imposed	C1
Initial particle images (no.)	478,036
Final particle images (no.)	172,639
Map resolution (Å)	2.9
FSC threshold	0.143
Map resolution range (Å)	2.6 to 5
**Refinement**	
Initial model used (PDB code)	7T9I
Model composition in the ASU	
Non-hydrogen atoms	7810
Protein residues	993
Ligands	37
B factors ($Å^{2}$)	
Protein	86.2
Ligand	122.8
R.m.s. deviations	
Bond lengths (Å)	0.0078
Bond angles (°)	0.77
Validation	
MolProbity score	1.84
Clash score	4.17
Poor rotamers (%)	2.24
Ramachandran plot	
Favoured (%)	95
Allowed (%)	5
Disallowed (%)	0

Supplementary Table 2 | Numerical values for data displayed in the bar graphs Figs 3b-d

	E_{max}				pEC_{50}				Cell surface expression (CSE)			
	E_{max} (%)	SD	t-test (p value)	n	pEC_{50}	SD	t-test (p value)	n	CSE (%)	SD	t-test (p value)	n
M1	100	14.1	n/a	6	5.07	0.07	n/a	6	40.0	11.3	n/a	4
D127A	17.4	7.8	<0.0001	6	4.53	0.52	0.0511	6	31.7	29.0	0.62	4
V128A	86.5	3.5	0.0572	6	4.38	0.08	<0.0001	6	23.4	7.3	0.11	2
C131A	77.7	6.0	0.0085	6	4.16	0.05	<0.0001	6	33.7	15.0	0.58	3
Y132A	-0.7	6.7	<0.0001	5	-	-	-	5	58.7	26.6	0.50	2
N217A	96.3	5.4	0.5020	6	4.41	0.08	<0.0001	6	49.8	16.6	0.44	3
W286Y	2.0	1.1	<0.0001	6	-	-	-	6	45.0	21.4	0.70	4
Y289A	132.6	31.9	0.0605	6	3.55	0.26	<0.0001	6	32.7	23.8	0.61	4
F290A	-1.4	4.0	<0.0001	3	-	-	-	3	0.0	0.0	0.06	3
V312A	151.1	11.1	<0.0001	6	4.90	0.12	0.0164	6	39.6	25.1	0.98	3
V315A	0.7	2.5	<0.0001	4	-	-	-	4	30.0	18.0	0.39	4
Y316A	10.6	2.1	<0.0001	6	3.48	0.17	<0.0001	6	34.9	23.1	0.75	4

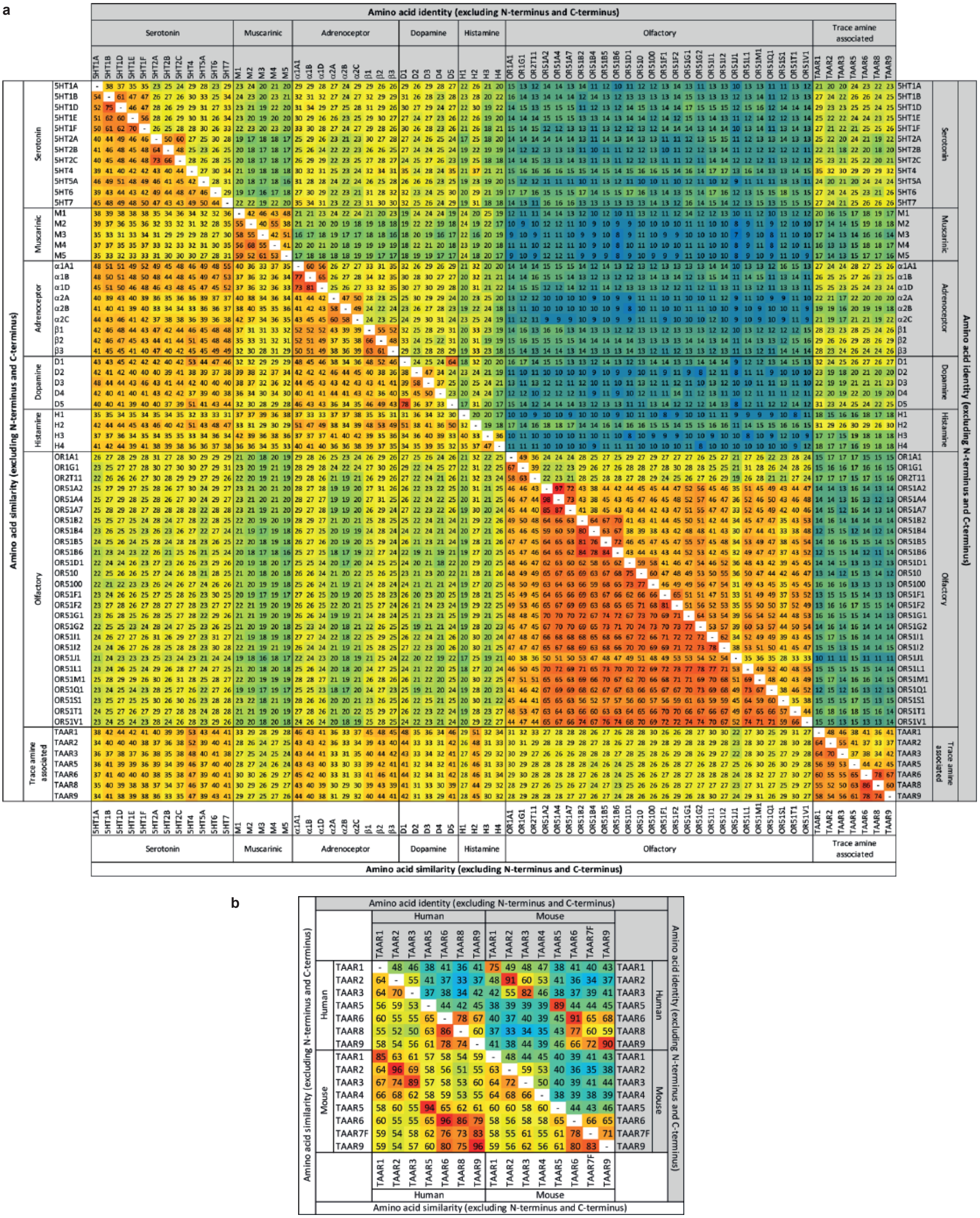

Supplementary Fig. 1 | Comparison of receptor amino acid sequences. a, Comparison of amino acid sequence identity (top right quadrant) and similarity (bottom left quadrant) of human aminergic receptors and a selection of human odorant receptors (as defined in GPCRdb). **b**, Comparison of amino acid sequence identity (top right quadrant) and similarity (bottom left quadrant) of mouse TAARs and human TAARs. In both panels, alignments excluded the N-terminus and C-terminus of receptors which are the most variable regions of receptors and do not impact the structure of the trans-membrane α-helices as the termini form unstructured regions. Figures were prepared using alignments and data generated by GPCRdb. Rainbow coloration spans from the least similar/identical receptors in blue to the most similar/identical receptors in red.

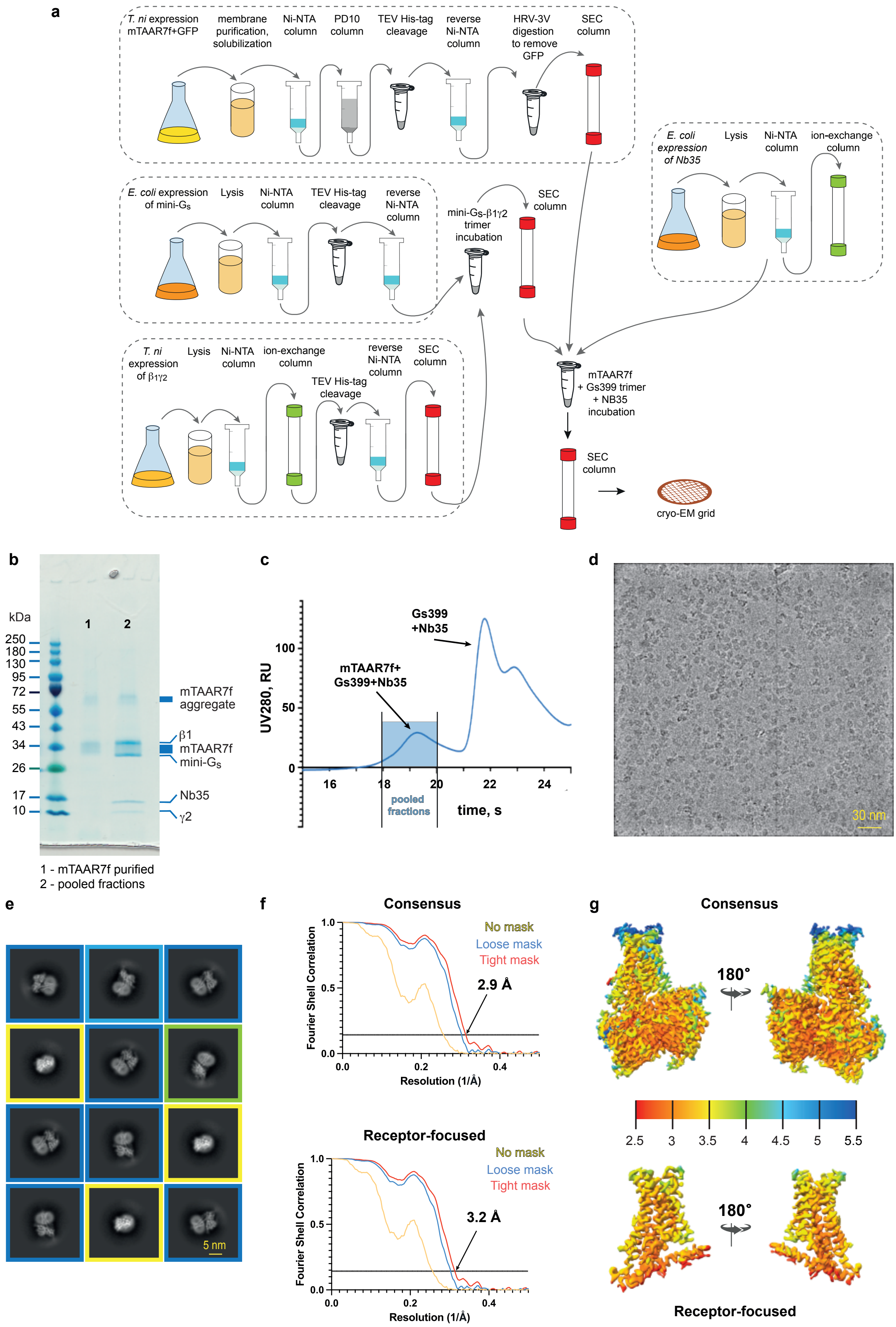

Supplementary Fig. 2 | mTAAR7f purification and cryo-EM of the mTAAR7f–mini-Gs–Nb35 complex. a, Purification scheme for the preparation of the mTAAR7f–miniGs–Nb35 complex for structure determination by cryo-EM. **b**, Coomassie Blue-stained SDS-PAGE gel of purified mTAAR7f (lane 1) and pooled fractions of the mTAAR7f–miniGs–Nb35 complex after gel filtration (lane 2). Individual components are indicated. **c**, Gel filtration trace of the mixture of mTAAR7f with miniGs and Nb35. **d**, A representative cryo-EM micrograph (defocus −2.4 μm) from the collected dataset. **e**, Representative 2D class averages of the mTAAR7f–miniGs–Nb35 complex determined using the initial set of particles following several rounds of 2D classification. Class averages corresponding to similar particle orientations are marked with the same coloured frames: blue, side views; green, partial side view; yellow, top views. **f**, FSC curves of the receptor focused and consensus reconstructions show an overall resolution of 3.2 Å and 2.9 Å, respectively, using the gold standard FSC of 0.143. **g**, Local resolution estimation of the receptor-focused and consensus maps of the mTAAR7f–miniGs–Nb35 as calculated by CryoSparc.

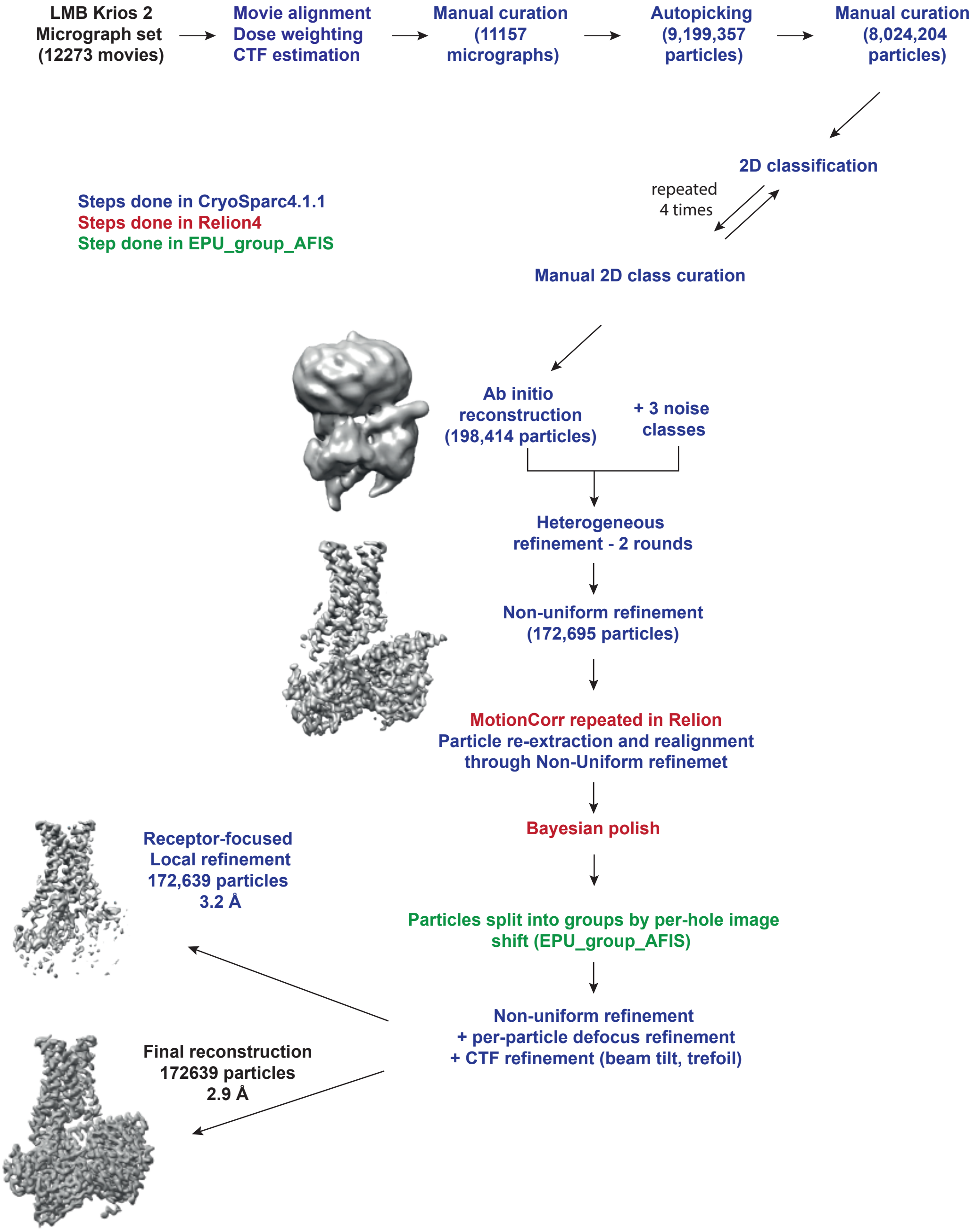

Supplementary Fig. 3 | Flow chart of cryo-EM data processing. The dataset was collected in one session (48 h) on the LMB Krios 2 equipped with Falcon 4 detector. The movies were corrected for drift, beam-induced motion and radiation damage using CryoSparc motion correction implementation. After estimation of CTF parameters, the dataset was manually curated to exclude low quality micrographs. Particles were picked using a Gaussian blob and subjected to four rounds of 2D classification, after each round only species resembling a receptor-G protein complex were retained. Particles in the best 2D classes were subjected to two rounds of heterogenous refinement in CryoSparc versus three separately generated classes corresponding to picks without any structural features (noise classes). The output particles were subjected to one round of non-uniform refinement in CryoSparc resulting in a global resolution of 3.05 Å. To perform post-processing steps in Relion, motion correction was repeated in Relion followed by particle re-extraction and realignment. Bayesian polishing was performed in Relion, and particles were also split into AFIS groups using EPU_group_AFIS script. The final step of non-uniform refinement coupled to per-particle defocus refinement and per-particle CTF refinement, including beam-tilt, trefoil and tetrafoil corrections, was performed in CryoSparc. The final model based on 172,639 particles achieved a global resolution of 2.9 Å, while the receptor-focused map achieved a global resolution of 3.2 Å. Resolution of the models after refinements was calculated with the gold-standard FSC of 0.143 in CryoSparc (Supplementary Fig. 2f).

a

Receptor	Receptor state	Binding partner	PDB code	Ligand name	Ligand type	TM2				ECL1	TM3									TM4	ECL2						TM5					TM6					TM7							
						2.53	2.61	2.64	2.65	23.50	3.25	3.28	3.29	3.32	3.33	3.35	3.36	3.37	3.40	4.56	N/A	N/A	45.50	45.51	45.52	N/A	5.38	5.39	5.42	5.43	5.46	6.48	6.51	6.52	6.55	6.58	7.31	7.32	7.35	7.36	7.39	7.40	7.42	7.43
mTAAR7f	Active	Gs	-	DMCHA	Ag									D			C	Y											N			W	Y								V		V	Y
hβ_2AR	Inactive states	None	3D4S	Timolol	Ant	M						W	T	D	V		V	T							F		Y	A	S	S	S	W	F	F	N				Y		N			Y
			3NY8	CHEMBL1233766	Ant	M						W	T	D	V		V	T							F		Y		S	S	S	W	F	F	N				Y		N			Y
			3NY9	CHEMBL1233771	Ant	M						W	T	D	V		V	T							F	T	Y	A	S	S	S	W	F	F	N				Y		N			Y
			3NYA	CHEMBL1160734	Ant	M						W	T	D	V		V	T							F		Y	A	S	S	S	W	F	F	N				Y		N			Y
			3PDS	FAUC50	Ag		G	C	I	W		W	T	D	V		V						C	D	F	T	Y	A	S	S	S	W	F	F	N				Y	I	N	W		Y
			5D5A	Carazolol	Ant							W	T	D	V		V	T							F	T	Y	A	S	S	S	W	F	F	N				Y		N			Y
			6PS3	(S)-Carvedilol	Ant		G	C	I			W	T	D	V		V	T						D	F	T	Y	A	S	S	S	W	F	F	N				Y	I	N	W		Y
			6PS5	Propranolol	Ant							W	T	D	V		V	T							F		Y	A	S	S	S	W	F	F	N				Y		N			Y
	Active states	G_s	3SN6	BI-167107	Ag							W	T	D	V		V	T					C	D	F		Y	A	S	S	S	W	F	F	N			K	Y	I	N			Y
		Nanobody	4LDE	BI-167107	Ag			C				W	T	D	V		V	T					C	D	F	T	Y	A	S	S	S	W	F	F	N				Y	I	N			Y
			4LDO	Adrenaline	Ag							W		D	V		V	T							F				S	S	S	W	F	F	N				Y		N			Y
			4LDL	Hydroxybenzylisoproterenol	Ag			C				W	T	D	V		V	T						D	F		Y		S	S	S	W	F	F	N			K	Y	I	N			Y
			4QKX	Q27453560	Ag		G	C	I	W		W		D	V		V	T					C	D	F		Y		S	S	S	W	F	F	N				Y	I	N	W		Y
			6MXT	Salmeterol	Ag			C				W	T	D	V		V	T						D	F	T	Y	A	S	S	S	W	F	F	N	H	R	K	Y	I	N			Y
		G_s	7BZ2	Formoterol	Ag			C				W	T	D	V		V	T						D	F	T	Y	A	S	S	S		F	F	N				Y		N			Y
			7DHI	Levosalbutamol	Ag							W	T	D	V		V	T							F		Y		S	S	S	W	F	F	N				Y		N			Y
			7DHR	(R)-Isoproterenol	Ag							W	T	D	V		V	T							F		Y		S	S	S	W		F	N				Y		N			Y

b

	Receptor (% identity)	TM3			TM5	TM6		TM7		
		3.32	3.36	3.37	5.42	6.48	6.51	7.39	7.42	7.43
	**mTAAR7f (100%)**	D	C	Y	N	W	Y	V	V	Y
Human TAARs	hTAAR9 (71.5%)	D	C	F	C	W	Y	V	V	Y
	hTAAR6 (67.4%)	D	C	Y	D	W	Y	C	A	Y
	hTAAR8 (66.0%)	D	C	Y	D	W	Y	C	A	Y
	hTAAR5 (46.0%)	D	C	L	N	W	F	I	A	Y
	hTAAR3 (44.3%)	D	R	L	L	W	C	V	R	Y
	hTAAR1 (41.6%)	D	S	S	T	W	F	I	G	Y
	hTAAR2 (38.2%)	D	S	I	L	W	F	T	G	Y
Mouse TAARs	mTAAR7e (93%)	D	C	S	N	W	Y	V	A	Y
	mTAAR7d (91%)	E	C	Y	N	W	Y	V	V	Y
	mTAAR7b (90%)	D	C	Y	N	W	Y	V	A	Y
	mTAAR7a (90%)	E	C	Y	N	W	Y	V	A	Y
	mTAAR8c (69%)	D	C	Y	S	W	Y	C	A	Y
	mTAAR8b (70%)	D	C	Y	D	W	Y	C	A	Y
	mTAAR9 (69%)	D	C	F	C	W	Y	V	V	Y
	mTAAR8a (68%)	D	C	F	C	W	Y	V	V	Y
	mTAAR6 (68%)	D	C	Y	D	W	Y	V	A	Y
	mTAAR5 (44%)	D	C	L	N	W	F	I	A	Y
	mTAAR3 (44%)	D	S	L	L	W	C	V	G	Y
	mTAAR1 (42%)	D	C	L	N	W	F	I	A	Y
	mTAAR2 (38%)	D	S	I	L	W	C	T	G	Y
	mTAAR4_incomplet	D	C	T	A	W	F	L	G	Y

c

Receptor (% identity)	DRY motif			Transm. Switches					NPxxY motif		
	3.49	3.50	3.51	3.36	3.40	5.50	6.44	6.48	7.49	7.50	7.53
mTAAR7f (100%)	D	R	Y	C	L	P	F	W	N	P	Y
hTAAR9 (71.5%)	D	R	Y	C	L	P	F	W	N	P	Y
hTAAR6 (67.4%)	D	R	Y	C	L	P	F	W	N	P	Y
hTAAR8 (66.0%)	D	R	Y	C	V	P	F	W	N	P	Y
hTAAR5 (46.0%)	D	R	H	C	I	P	Y	W	N	P	Y
hTAAR3 (44.3%)	D	R	F	R	I	P	F	W	N	P	H
hTAAR1 (41.6%)	D	R	Y	S	I	P	F	W	N	P	Y
hTAAR2 (38.2%)	D	R	F	S	I	P	F	W	N	P	Y

Supplementary Fig. 4 | Amino acid sequence conservation in the OBS and activation switches of TAARs and β_2AR. a, Amino acid residues within 3.9 Å of ligands in the mTAAR7f structure and structures of human β_2AR. **b**, Amino acid residues within 3.9 Å of ligands in the mTAAR7f structure aligned with the equivalent residues in both human and mouse TAARs. Sequences were aligned using Clustal Omega[57,58] and the percentage of the full-length receptor sequence identity to mTAAR7f was calculated using the web-based resource BLAST[59]. **c**, Conservation in TAARs of the D-R-Y motif, transmission switches (including the P-I-F motif, marked in red) and N-P-x-x-Y motifs. Sequences were aligned using Clustal Omega[57,58]. Percentage of the full-length receptor sequence identity to mTAAR7f was calculated using web-based resource BLAST[59]

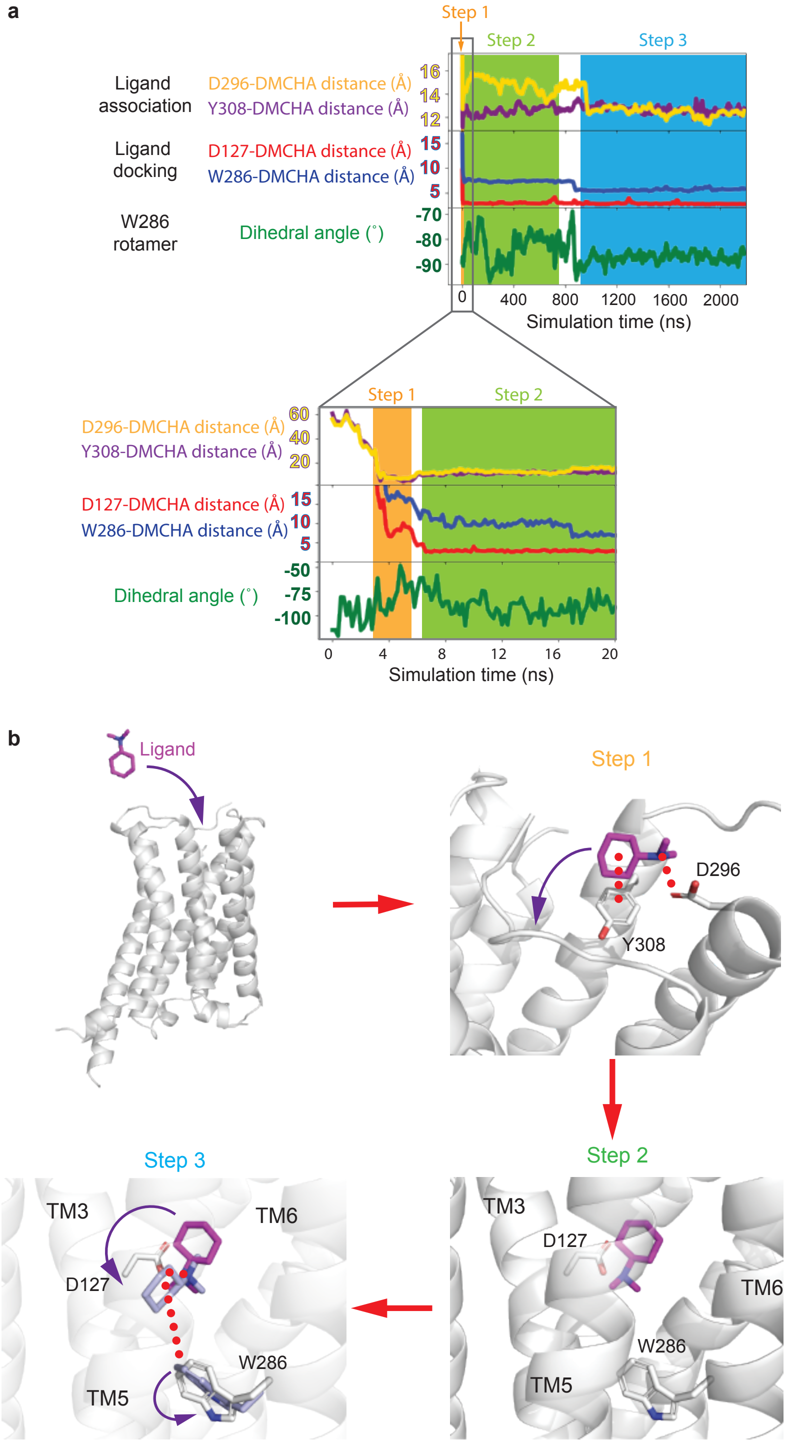

Supplementary Fig. 5 | MD simulation of DMCHA association to mTAAR7f. a, Four 2.2 μsec velocity MD simulations were performed on mTAAR7f (no G protein) in the presence of ligand outside the OBS. One trajectory is shown here where the ligand remained stably associated with the receptor at the end of the simulation, and three further examples are shown in Supplementary Fig. 8, and the variation of backbone atom and ligand atom RMSD during the simulations is shown in Supplementary Fig. 9. The ligand was observed to enter the OBS rapidly. Ligand clustering analysis identified specific residues (Asp296$^{6.58}$ and Tyr308$^{7.35}$) that associated with DMCHA upon initial association with the receptor. The process is plotted visually through measuring distances between DMCHA and the residues in the extracellular region (Asp296$^{6.58}$ and Tyr308$^{7.35}$) and in the OBS (Asp127$^{3.32}$ and Trp286$^{6.48}$). The motion of Trp286$^{6.48}$ is monitored through the variation in its Chi2 angle. **b**, Three-step model for the binding of DMCHA into mTAAR7f. Note that this simulation was performed on mTAAR7f in an active state and might not represent fully the trajectory in an inactive state in the absence of a G protein. However, given our understanding of the role of the G protein in closing the entrance of the OBS and decreasing its volume upon G protein coupling in the βARs, then the data here may represent an underestimate of the rate of ligand association.

```
                              .         .         .         .         .         .
mTAAR7f     1 MSIADETVSWNQDSILSRDLFSATSAELCYENLNRSCVRSPYSPGPRLILYAVFGFGAVL  60
hADRB2      1               MGQPGNGSAFLLAPNGSHAPDHDVTQERDEVWVVGMGIVMSLIVLA  46
                         .      **        .     *          . .   *     .

                  1x50 .         .         .  2x50   .         .         .
mTAAR7f    61 AVCGNLLVMTSILHFRQLHSPANFLVASLACADFLVGVMVMPFSMVRSVEGCWYFGDSYC 120
hADRB2     47 IVFGNVLVITAIAKFERLQTVTNYFITSLACADLVMGLAVVPFGAAHILMKMWTFGNFWC 106
               * **.**.*.*  .*  .*..  .*.  ..******  ..*.  *.**     . .   * **   *

                         .         .      3x50 .         .         . 4x50    .
mTAAR7f   121 KLHTCFDVSFCYCSLFHLCFISVDRYIAVSDPLAYPTRFTASVSGKCITFSWLLSISYGF 180
hADRB2    107 EFWTSIDVLCVTASIETLCVIAVDRYFAITSPFKYQSLLTKNKARVIILMVWIVSGLTSF 166
              -  *.  **     .*.   **  *.****  *..  *  *  .   *     .   *    *..*   *

                         .         .         .          .   5x50  .
mTAAR7f   181 SLIYTGASEAGLEDLVSSLTCVGGCQIAVNQTWVFIN-FSVFLIPTLVMITVYSKIFLIA 239
hADRB2    167 LPIQMHWYRATHQEAINCYANETCCDFFTNQAYAIASSIVSFYVPLVIMVFVYSRVFQEA 226
                *       *    .. . . .    *      **.        *  .*  ..*.  ***..*   *

              .         .         .                       .         .
mTAAR7f   240 KQQAQNIEKMSKQTARASDSYKDRVAKR-------------ERKAAKTLGIAVAAFLLSW 286
hADRB2    227 KRQLQKIDKSEGRFHVQNLSQVEQDGRTGHGLRRSSKFCLKEHKALKTLGIIMGTFTLCW 286
              *.* * *.*    .      *     .. .              *.** *****  . .* *.*

                 6x50          .         .         .   7x50    .         .
mTAAR7f   287 LPYFIDSFIDAFLGFITPTYVYEILVWIVYYNSAMNPLIYAFFYPWFRKAIKLTVTGKIL 346
hADRB2    287 LPFFIVNIVHVIQDNLIRKEVYILLNWIGYVNSGFNPLIYCRSPDFRIAFQELLCLRRSS 346
              **.**     .      .    **  .*  ** *  **   *****.          *       .

                 .
mTAAR7f   347 RENSSTTNLFSE                                                 358
hADRB2    347 LKAYGNGYSSNGNTGEQSGYHVEQEKENKLLCEDLPGTEDFVGHQGTVPSDNIDSQGRNC 406
                  .

mTAAR7f   359         358
hADRB2    407 STNDSLL 413
```

Supplementary Fig. 6 | Alignment of the amino acid sequences of mTAAR7f and β2AR. Red bars, transmembrane regions; yellow bar, amphipathic helix 8; red residues, Ballesteros Weinstein numbering system xx.50. The alignment was performed using the program MacVector.

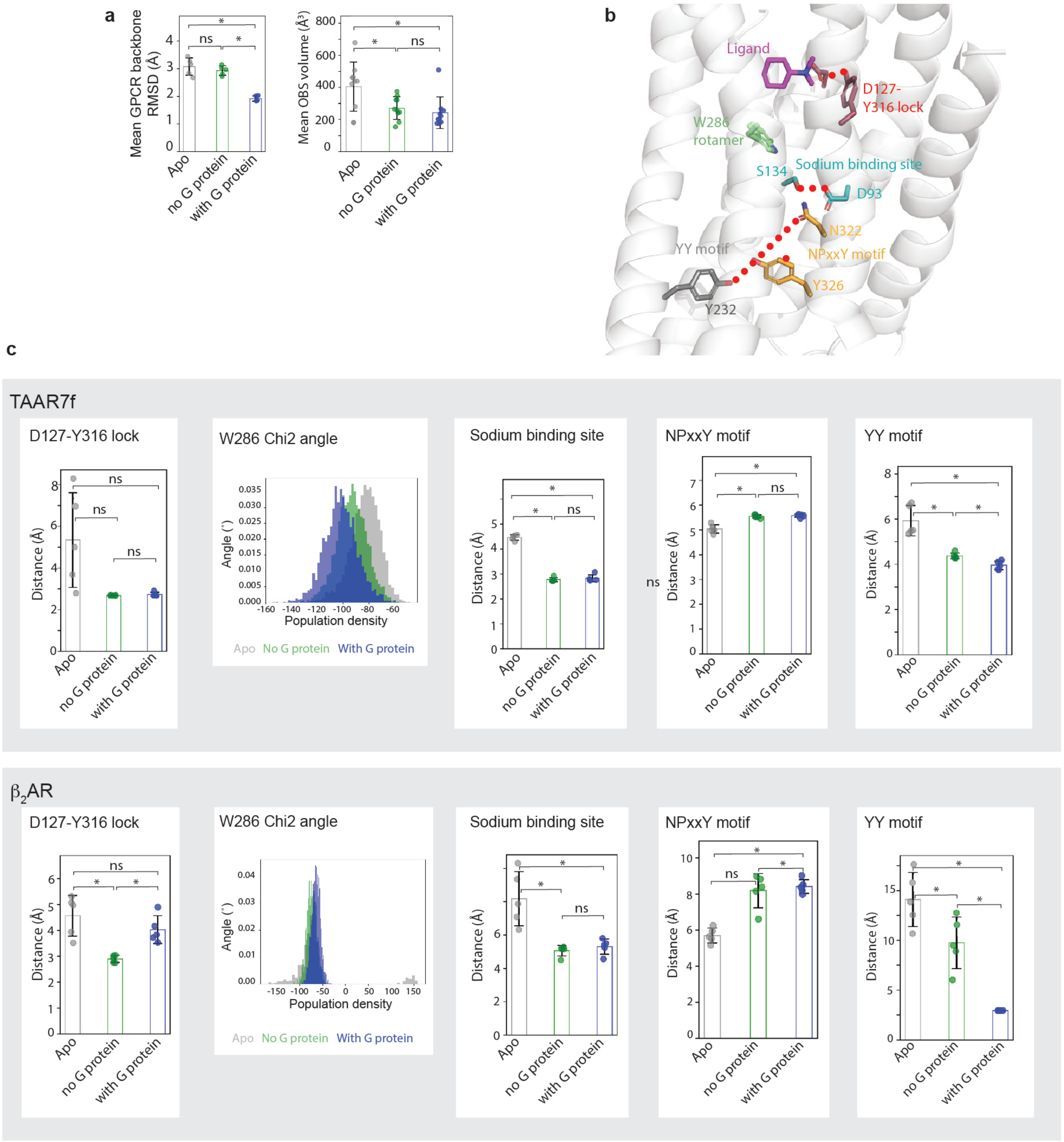

Supplementary Fig. 7 | MD simulations of mTAAR7f and analysis of changes in activation switches. a, Five independent MD simulations were performed either on the mTAAR7f-mini-G_S-DMCHA complex (with G protein), on mTAAR7f-DMCHA (no G protein) or on mTAAR7f alone (Apo). The mean GPCR backbone RMSD and the mean volume of the OBS are plotted for each simulation and found to increase significantly in the Apo simulations compared to when G protein and ligand are bound. **b**, Position of the transmission elements in mTAAR7f that were analysed to assess whether the receptor was remaining in the state defined by the cryo-EM structure. These included all the canonical transmission switches in Class A GPCRs. **c**, For each of the five independent simulations distances were plotted between residues that define the state of the transmission switches. No significant differences were observed in the OBS ($D127^{3.32}$-$Y316^{7.43}$ lock), but increases in distances were observed for both mTAAR7f and β2AR in the YY motif and the sodium binding site, consistent with a tendency towards a more inactive state. No change was observed in the NPxxY motif in mTAAR7f, but β2AR changed towards a more inactive state. Changes in Chi2 angle of $Trp286^{6.48}$ in mTAAR7f show a tendency towards a more inactive state in the Apo simulation, but this is not in evidence in β2AR. Data for simulations on β2AR were obtained from GPCRmd. The error bars represent the SD and a t-test showed either no statistical difference (ns) or a statistical difference (*, $p < 0.05$) between data. The variation of backbone atom and ligand atom RMSD during the simulations is shown in Supplementary Fig. 9. See Source Data for all numerical values.

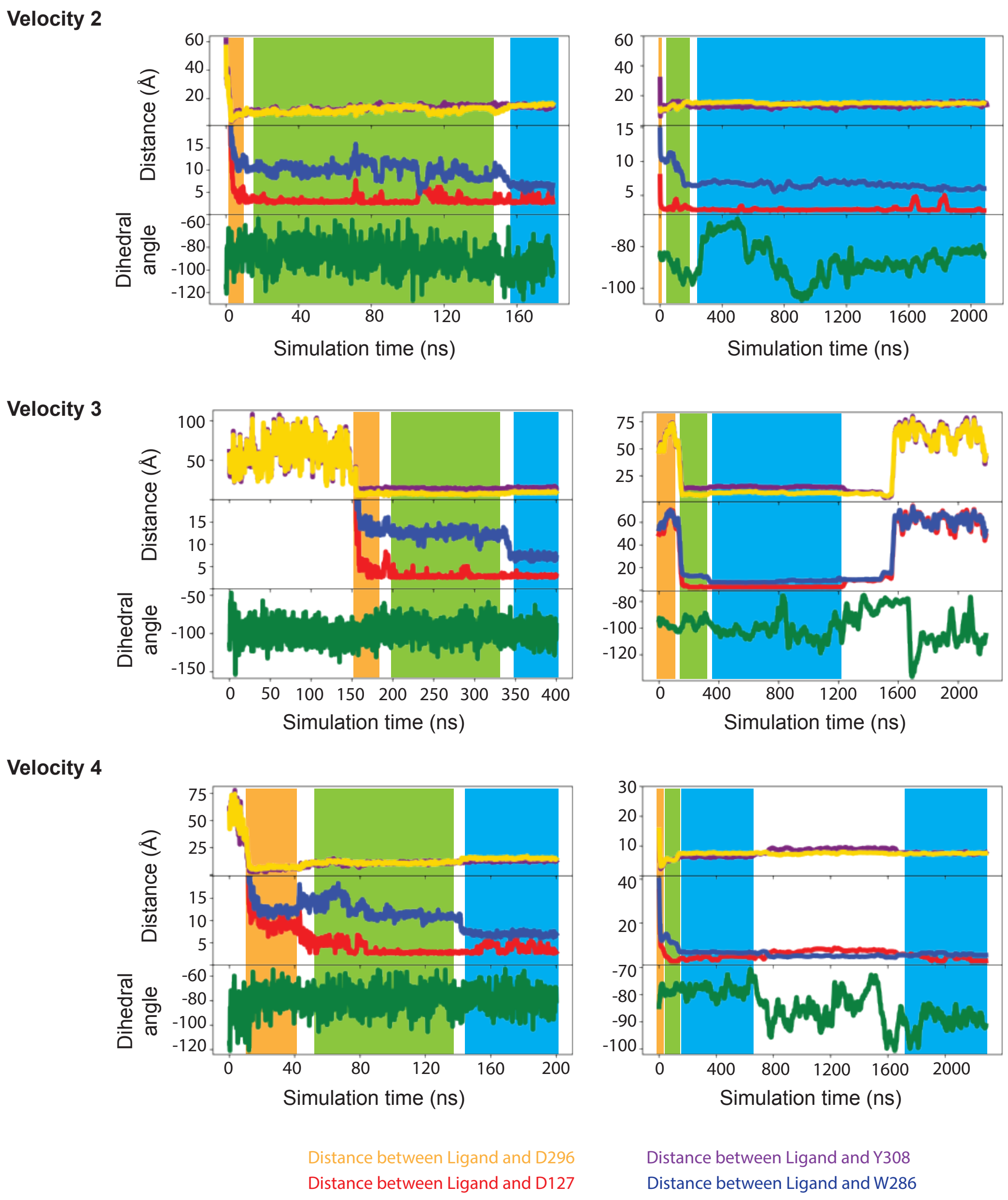

Supplementary Fig. 8 | MD simulation of DMCHA association to mTAAR7f. Three additional 2.2 μsec velocity MD simulation are shown of mTAAR7f (no G protein) in the presence of ligand outside the OBS. The colour scheme is identical to that in Supplementary Fig. 5a. The blue area in the traces (Step 3) represents where the simulated position of DMCHA is similar to that in the cryo-EM structure. In Velocity 2 the ligand is stable in Step 3, but in Velocity 3 the ligand dissociates and does not re-bind. In Velocity 4 the DMCHA initially adopts a pose similar to the cryo-EM structure, but then rotates away from it, until re-adopting the cryo-EM pose 1 μsec later. Panels on the left represent the first 200-400 nsec and the panels on the right show the whole 2.2 μsec simulation. The variation of backbone atom and ligand atom RMSD during the simulations is shown in Supplementary Fig. 9.

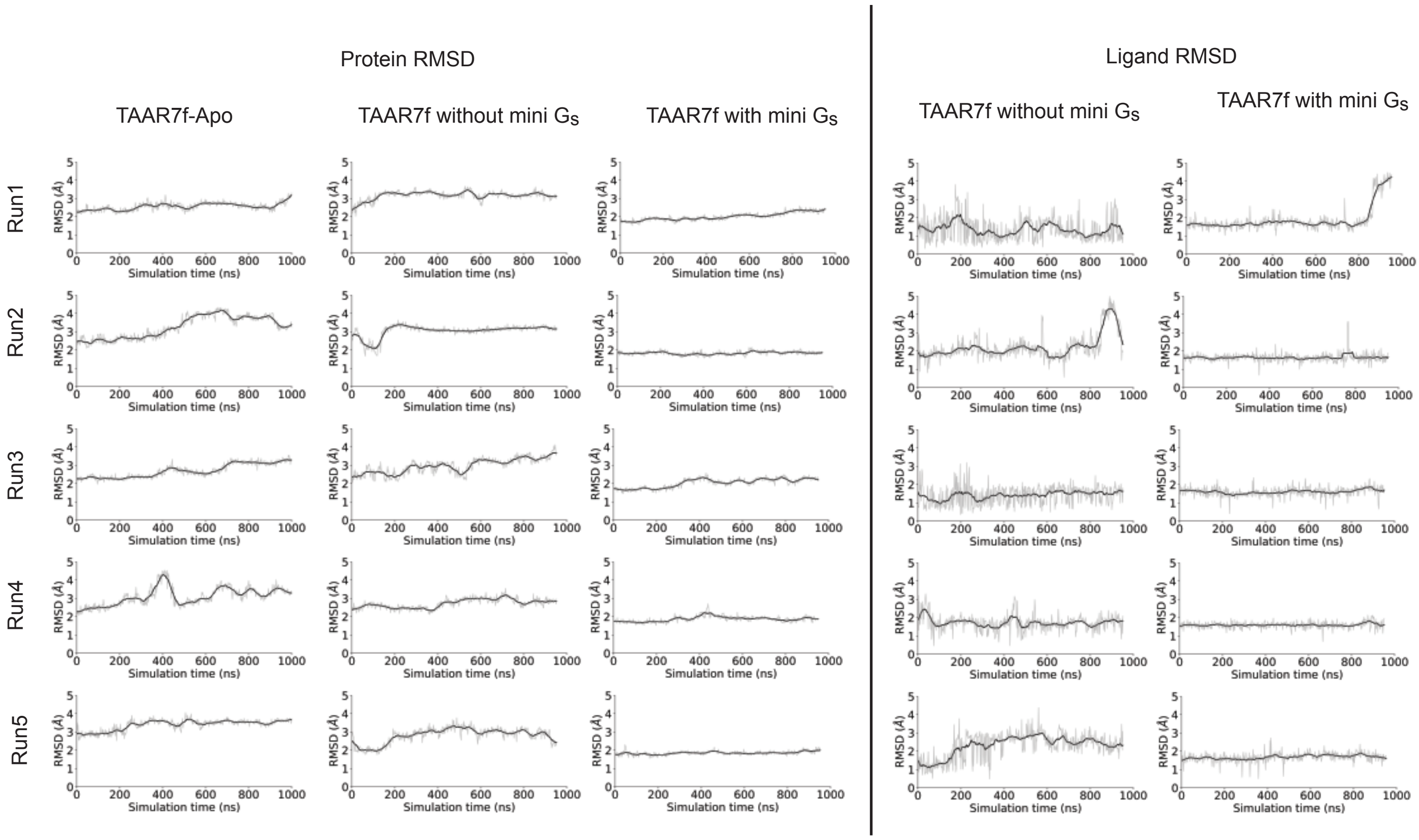

Supplementary Fig. 9 | Variation of backbone atom and ligand atom RMSD during MD simulations.
Raw data is plotted (grey line) along with a smoothing curve (black line; sliding window size = 50).
